# Supplementary material for: Genomic tailoring of autogenous poultry vaccines to reduce Campylobacter from farm to fork
Source: NPJ Vaccines. 2024 Jun 12;9:105. doi: 10.1038/s41541-024-00879-z (PMC11169640; doi:10.1038/s41541-024-00879-z)
Supplement: Supplementary file 1 — Supplementary Material [file 41541_2024_879_MOESM1_ESM.pdf]

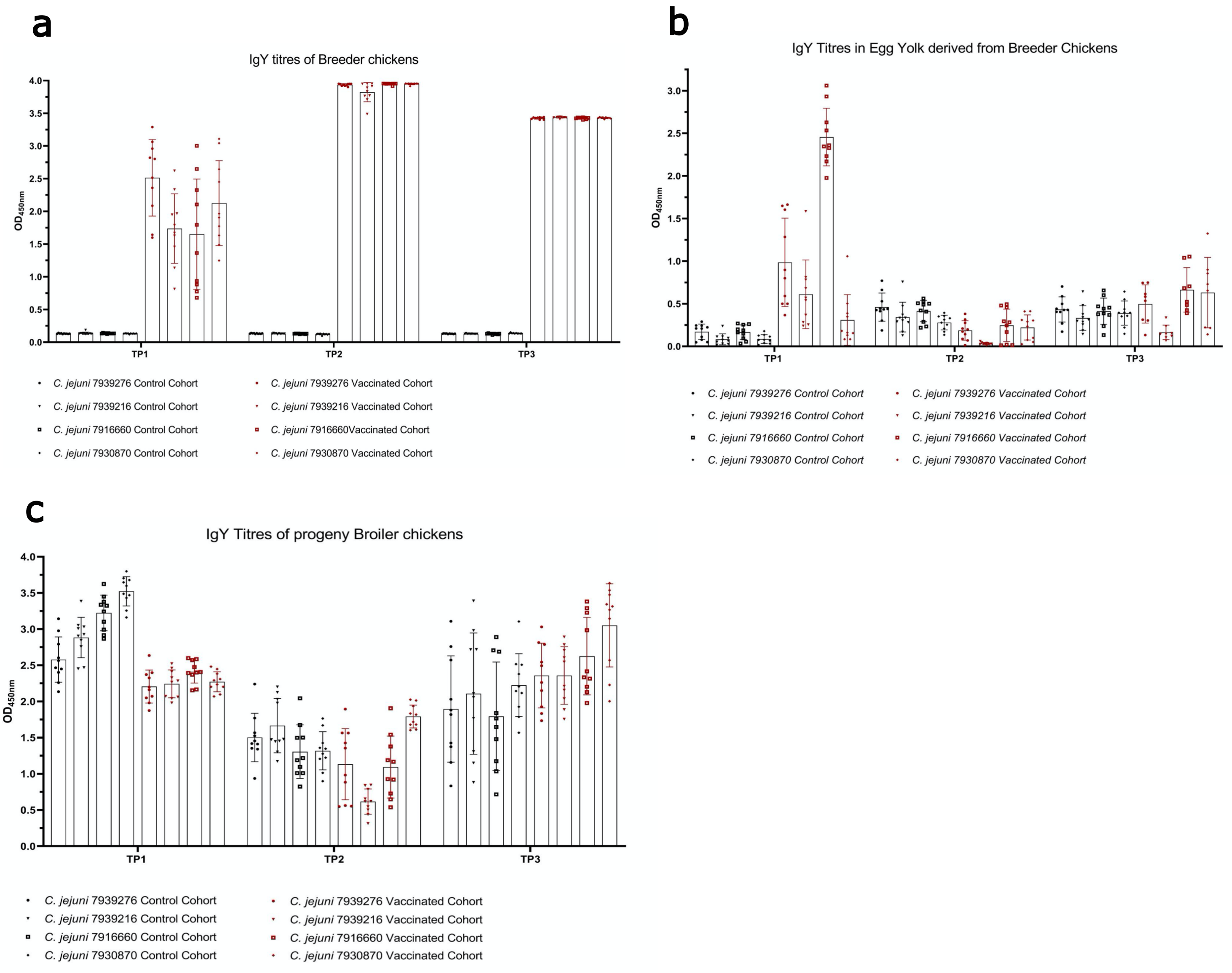

Supplementary Figure 1. Pre-vaccine isolate ELISA plots. Individual plots of IgY titres per vaccine isolate for breeder blood (a), breeder egg yolk (b) and broiler blood (c).

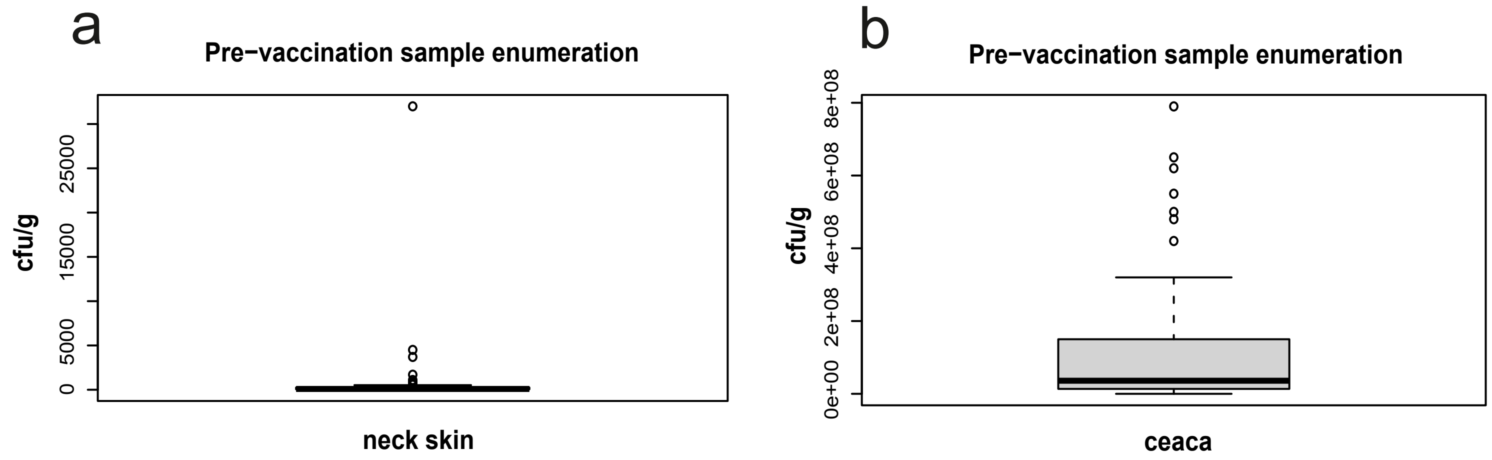

c

Pre-vac sampling- understanding strain diversity						
	Neck skin			ceaca		
	Avg. (cfu/g)	No. of samples collected	%Campylobacter +ve	Avg. (cfu/g)	No. of samples collected	%Campylobacter +ve
**Farm 1**	334	30	83	2.05E+08	30	100
**Farm 2**	32	30	27	2.95E+06	30	7
**Farm 3**	335	30	70	9.54E+07	30	73
**Farm 4**	50	30	63	0	30	0
**Farm 5**	1389	30	97	2.80E+07	30	97

Supplementary Figure 2. Pre-vaccination enumeration (raw). *Campylobacter* enumeration values per neck skin (a) and caeca (b) sample before the removal of neck skin outlier samples (>1000 cfu/g). The per farm enumeration for *Campylobacter* is also summarised (c).

Post-vaccination sample enumeration

a

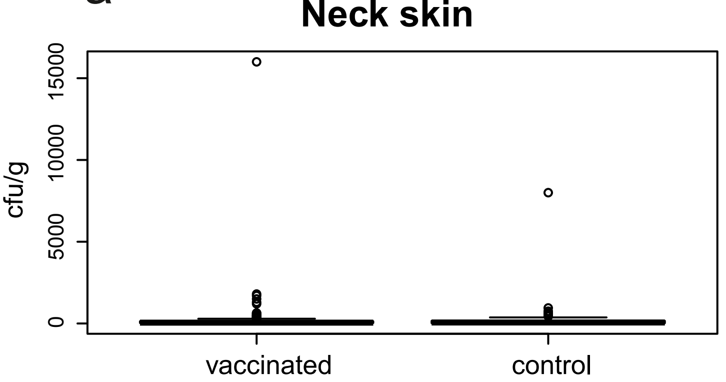

b

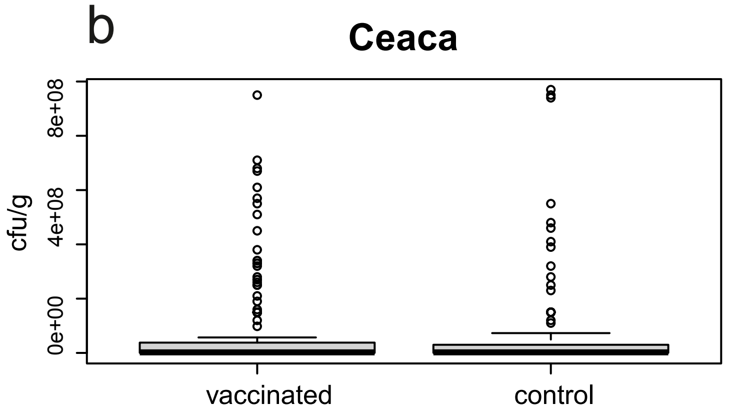

c

Post-vac sampling - unvaccinated (control) cohort						
	Neck skin			ceaca		
	Avg. (cfu/g)	No. of samples collected	%Campylobacter +ve	Avg. (cfu/g)	No. of samples collected	%Campylobacter +ve
**Farm 1**	662	30	53	3.18E+06	30	100
**Farm 2**	176	30	83	3.07E+07	30	100
**Farm 3**	113	30	40	8.18E+05	30	100
**Farm 4**	100	30	87	1.69E+08	30	70
**Farm 5**	47.5	30	27	1.53E+08	30	100

d

Post-vac sampling - Vaccinated cohort						
	Neck skin			ceaca		
	Avg. (cfu/g)	No. of samples collected	Campylobacter +ve	Avg. (cfu/g)	No. of samples collected	Campylobacter +ve
**Farm 1**	77	60	70	9.60E+06	60	100
**Farm 2**	3626	60	98	2.40E+07	60	98
**Farm 3**	128	60	45	1.17E+06	60	83
**Farm 4**	113	60	33	1.83E+08	60	77
**Farm 5**	57	60	50	1.19E+08	60	97

Supplementary Figure 3. Post-vaccination enumeration (raw). *Campylobacter* enumeration values per vaccinated and control neck skin (a) and caeca (b) samples before the removal of neck skin outlier samples (>1000 cfu/g). The per farm enumeration for *Campylobacter* is summarised for the control (c) and vaccinated (d) cohorts.

a odds ratio 3 = theta 1.099.

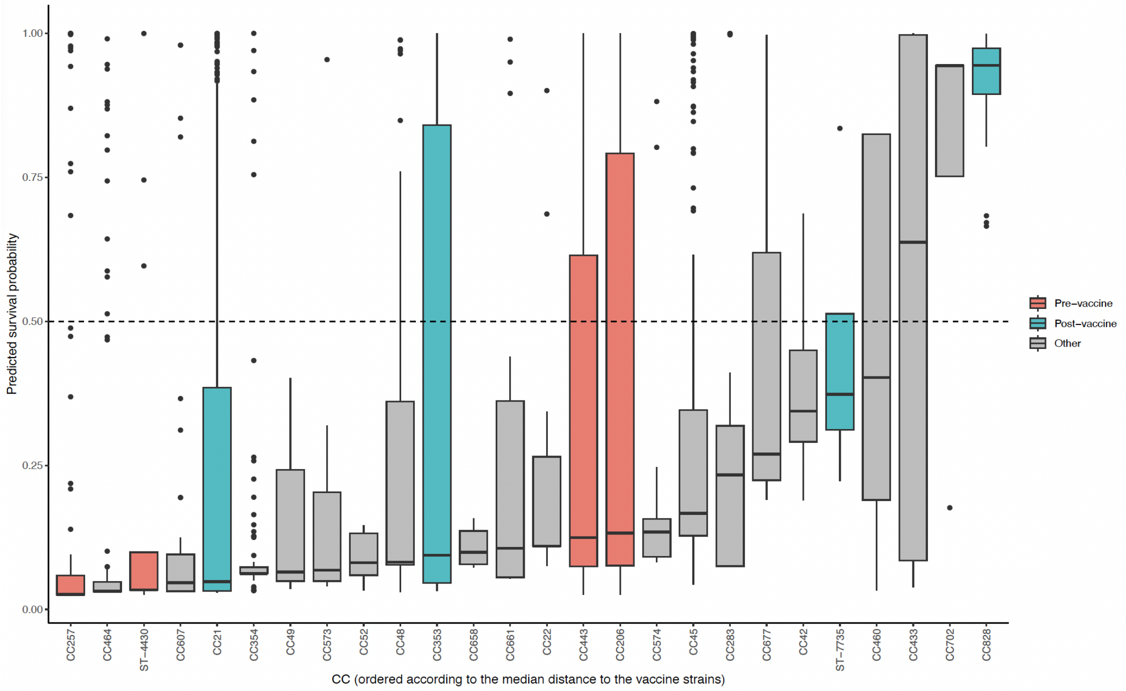

b odds ratio 2 = theta 0.693.

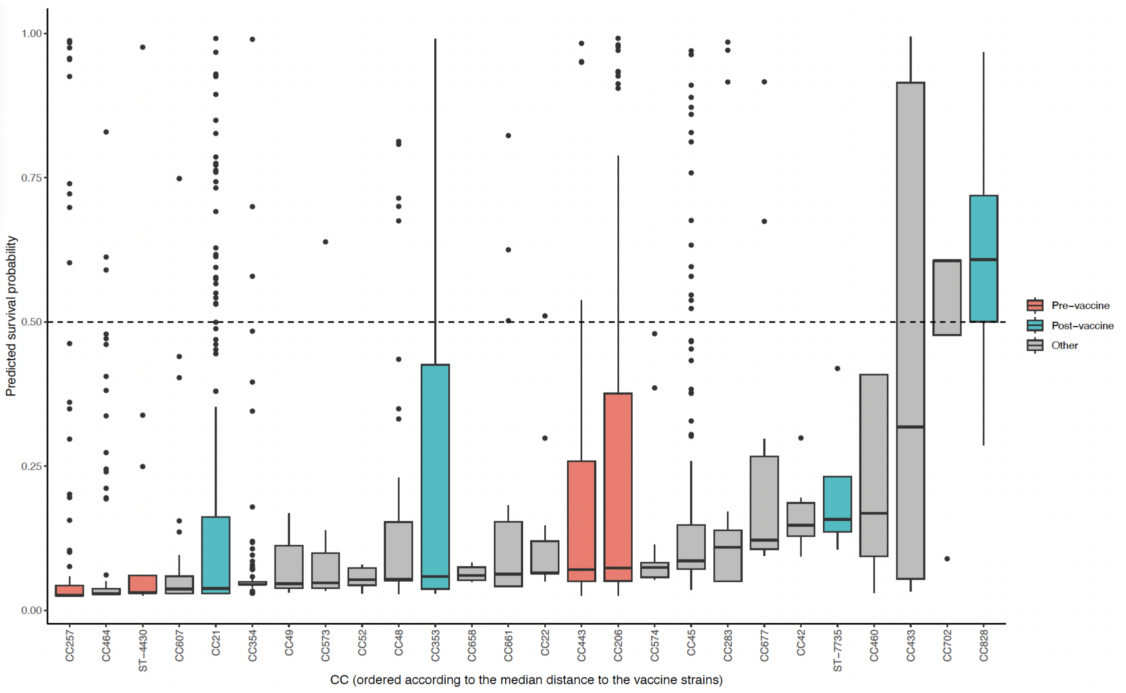

Supplementary Figure 4. Other odds ratios. The predicted survival probability of each chicken-associated CC using theta 1.099 (OR 3) (a) and theta 0.693 (OR 2) (b). Black horizontal lines per box plot represent the median survival probability for the CC. CCs along the x axis are ordered by median survivability of the effects of the vaccines (left = less likely to survive the vaccine, right = more likely to survive the vaccine). the dashed horizontal line across the whole plot represents the minimum probability at which survival post-vaccine is likely to occur. Red box plots are the CCs found in pre-vaccine sampling, blue box plots are the CCs found in post-vaccination sampling and grey box plots are other chicken-associated CCs included as context. Bounds of the boxes are the first and third quartiles. Whiskers represent the upper and lower bounds of the interquartile range. Data beyond these points are plotted as individual black dots.

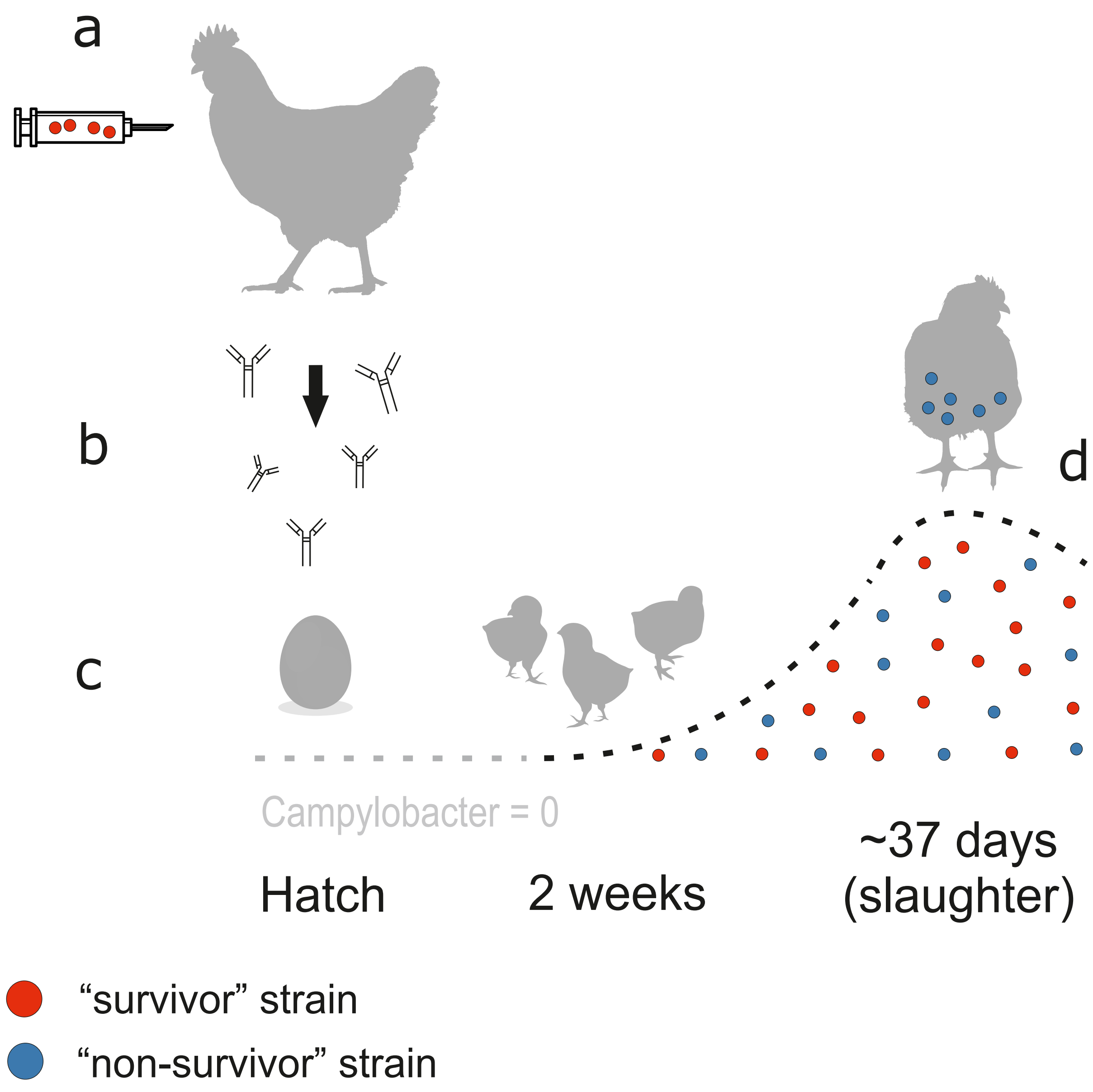

Supplementary Figure 5. Vaccine design based on protective effects of maternal antibody. Autogenous vaccine target: "survivor" isolates. Approximately 40,000 breeder chickens from a whole farm in Norfolk, UK were immunised with a vaccine containing 4 survival-associated isolates (red circles) (a). Protection of progeny directed at the vaccine isolates passed down by vaccine-specific maternal antibodies to eggs for the first two weeks post-hatch (b). Broilers are not colonised with *Campylobacter* for the first two weeks of life due to maternal antibody protection (c). As protection is offered against isolates most likely to survive poultry processing, there is opportunity for broilers to be colonised with isolates less likely to survive (blue circles) due to natural competition in the gut (d).

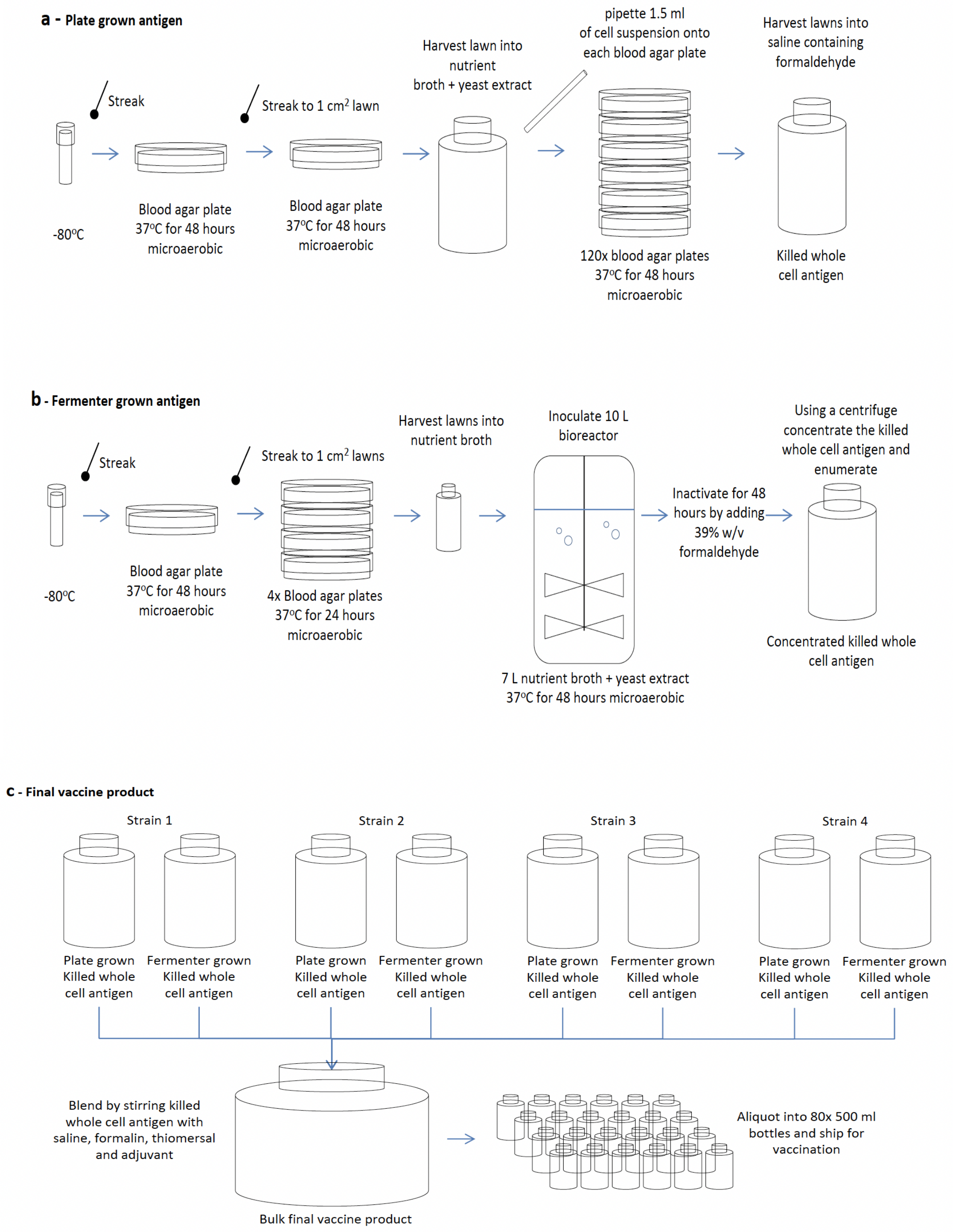

Supplementary Figure 6. Autogenous vaccine production. Illustration describing the production of the autogenous vaccine using the plate growth method (a), the fermenter growth method (b) and the combination of product from the two methods into the finished vaccine product (FVP) (c).

Supplementary Table 1. Raw enumeration summaries

Pre-vac sampling- understanding strain diversity

	Neck skin			ceaca		
	Avg. (cfu/g)	No. of samples collected	% Campylobacter +ve	Avg. (cfu/g)	No. of samples collected	% Campylobacter +ve
**Farm 1**	334	30	83	2.05E+08	30	100
**Farm 2**	32	30	27	2.95E+06	30	7
**Farm 3**	335	30	70	9.54E+07	30	73
**Farm 4**	50	30	63	0	30	0
**Farm 5**	1389	30	97	2.80E+07	30	97

Post-vac sampling - unvaccinated (control) cohort

	Neck skin			ceaca		
	Avg. (cfu/g)	No. of samples collected	% Campylobacter +ve	Avg. (cfu/g)	No. of samples collected	% Campylobacter +ve
**Farm 1**	662	30	53	3.18E+06	30	100
**Farm 2**	176	30	83	3.07E+07	30	100
**Farm 3**	113	30	40	8.18E+05	30	100
**Farm 4**	100	30	87	1.69E+08	30	70
**Farm 5**	47.5	30	27	1.53E+08	30	100

Post-vac sampling - Vaccinated cohort

	Neck skin			ceaca		
	Avg. (cfu/g)	No. of samples collected	Campylobacter +ve	Avg. (cfu/g)	No. of samples collected	Campylobacter +ve
**Farm 1**	77	60	70	9.60E+06	60	100
**Farm 2**	3626	60	98	2.40E+07	60	98
**Farm 3**	128	60	45	1.17E+06	60	83
**Farm 4**	113	60	33	1.83E+08	60	77
**Farm 5**	57	60	50	1.19E+08	60	97

Supplementary Table 2. Immune sera breeder titre dilution results

Dilution Factor		Neat	1:10	1:100	1:200	1:500
Immune Sera Breeder	1	OVR	OVR	OVR	2.95856	0.13546
	2	OVR	OVR	OVR	2.96415	0.13658
	3	OVR	OVR	OVR	2.92315	0.1357
	4	OVR	OVR	OVR	2.93584	0.13684
