# Supplementary material for: Genomic tailoring of autogenous poultry vaccines to reduce Campylobacter from farm to fork
Source: NPJ Vaccines. 2024 Jun 12;9:105. doi: 10.1038/s41541-024-00879-z (PMC11169640; doi:10.1038/s41541-024-00879-z)
Supplement: Supplementary file 2 — Reporting Summary [file 41541_2024_879_MOESM2_ESM.pdf]

nature portfolio

Corresponding author(s): Jessica K. Calland and Samuel K. Sheppard

Last updated by author(s): 2024/01/15

Reporting Summary

Nature Portfolio wishes to improve the reproducibility of the work that we publish. This form provides structure for consistency and transparency in reporting. For further information on Nature Portfolio policies, see our Editorial Policies and the Editorial Policy Checklist.

Statistics

For all statistical analyses, confirm that the following items are present in the figure legend, table legend, main text, or Methods section.

n/a	Confirmed	
☐	☒	The exact sample size (n) for each experimental group/condition, given as a discrete number and unit of measurement
☒	☐	A statement on whether measurements were taken from distinct samples or whether the same sample was measured repeatedly
☐	☒	The statistical test(s) used AND whether they are one- or two-sided<br>*Only common tests should be described solely by name; describe more complex techniques in the Methods section.*
☐	☒	A description of all covariates tested
☒	☐	A description of any assumptions or corrections, such as tests of normality and adjustment for multiple comparisons
☐	☒	A full description of the statistical parameters including central tendency (e.g. means) or other basic estimates (e.g. regression coefficient) AND variation (e.g. standard deviation) or associated estimates of uncertainty (e.g. confidence intervals)
☒	☐	For null hypothesis testing, the test statistic (e.g. F, t, r) with confidence intervals, effect sizes, degrees of freedom and P value noted<br>*Give P values as exact values whenever suitable.*
☒	☐	For Bayesian analysis, information on the choice of priors and Markov chain Monte Carlo settings
☒	☐	For hierarchical and complex designs, identification of the appropriate level for tests and full reporting of outcomes
☒	☐	Estimates of effect sizes (e.g. Cohen's d, Pearson's r), indicating how they were calculated

Our web collection on statistics for biologists contains articles on many of the points above.

Software and code

Policy information about availability of computer code

Data collection	No software was used specifically for data collection
Data analysis	Multiple sequence alignment building: MAFFT version 7<br>Phylogenetic tree building: fastTree version 2.1.8<br>Amino Acid Sequence Alignment: CLustal Omega version 1.2.4<br>Pairwise hamming distances: psdm version 0.1.0

For manuscripts utilizing custom algorithms or software that are central to the research but not yet described in published literature, software must be made available to editors and reviewers. We strongly encourage code deposition in a community repository (e.g. GitHub). See the Nature Portfolio guidelines for submitting code & software for further information.

Data

Policy information about availability of data

All manuscripts must include a data availability statement. This statement should provide the following information, where applicable:

- Accession codes, unique identifiers, or web links for publicly available datasets
- A description of any restrictions on data availability
- For clinical datasets or third party data, please ensure that the statement adheres to our policy

Assembled genomes and supplementary material are available from FigShare: doi: 10.6084/m9.figshare.25027232. Individual accession numbers can be found in Supplementary Tables 2 and 3.

Research involving human participants, their data, or biological material

Policy information about studies with human participants or human data. See also policy information about sex, gender (identity/presentation), and sexual orientation and race, ethnicity and racism.

Reporting on sex and gender	N/A
Reporting on race, ethnicity, or other socially relevant groupings	N/A
Population characteristics	N/A
Recruitment	N/A
Ethics oversight	N/A

Note that full information on the approval of the study protocol must also be provided in the manuscript.

Field-specific reporting

Please select the one below that is the best fit for your research. If you are not sure, read the appropriate sections before making your selection.

☒ Life sciences ☐ Behavioural & social sciences ☐ Ecological, evolutionary & environmental sciences

For a reference copy of the document with all sections, see nature.com/documents/nr-reporting-summary-flat.pdf

Life sciences study design

All studies must disclose on these points even when the disclosure is negative.

Sample size	No sample-size calculation was performed. However, multiple farms were sampled for the detection of Campylobacter.
Data exclusions	Outlier cfu/g counts >1000 were excluded for the neck skin samples for enumeration analysis. These were determined when analysing the distribution of values and determining 95% quantiles.
Replication	N/A
Randomization	Chickens were chosen randomised from each flock post-slaughter for removal of caeca and neck skin samples.
Blinding	N/A

Reporting for specific materials, systems and methods

We require information from authors about some types of materials, experimental systems and methods used in many studies. Here, indicate whether each material, system or method listed is relevant to your study. If you are not sure if a list item applies to your research, read the appropriate section before selecting a response.

Materials & experimental systems

n/a	Involved in the study	
☐	☒	Antibodies
☒	☐	Eukaryotic cell lines
☒	☐	Palaeontology and archaeology
☐	☒	Animals and other organisms
☒	☐	Clinical data
☒	☐	Dual use research of concern
☒	☐	Plants

Methods

n/a	Involved in the study	
☒	☐	ChIP-seq
☒	☐	Flow cytometry
☒	☐	MRI-based neuroimaging

Antibodies

Antibodies used	Anti-Chicken IgY (IgG) (whole molecule)–Peroxidase antibody produced in rabbit, Sigma Ltd,
Validation	Chicken IgY is the major antibody found in chicken serum. Chickens generate a comparatively enhanced antibody response to mammalian antigenic proteins. These immunoglobulins do not bind to Fc receptors present in mammals and bacteria, and also do not interact with rheumatoid factors. Thus anti-chicken antibodies are useful analytical tools for various research applications. Rabbit Anti-Chicken IgY (IgG) (whole molecule)-Peroxidase antibody binds to chicken IgG.

Animals and other research organisms

Policy information about studies involving animals; ARRIVE guidelines recommended for reporting animal research, and Sex and Gender in Research

Laboratory animals	N/A
Wild animals	N/A
Reporting on sex	Blood samples were obtained from male and female chickens. This was determined by a trained veterinarian. However, the sex of the chicken was not relevant for our study.
Field-collected samples	Samples were collected from broiler and breeder hens which were part of an existing poultry productions system. Birds were housed according to the standard procedures of the poultry company. Nothing was altered for the purpose of the study.
Ethics oversight	The Veterinary Medicines Directorate (VMD) oversaw and provided the license for the autogenous vaccine in this study. Blood, caeca and neck skin samples were obtained during routine practice and carried out or overseen by the prescribing veterinarian following regulations of the Veterinary Surgeons Act 1966.

Note that full information on the approval of the study protocol must also be provided in the manuscript.

Plants

Seed stocks	N/A
Novel plant genotypes	N/A
Authentication	N/A
